# Assessing Diet Quality Where Families Share Their Meals: Evidence from Malawi

**DOI:** 10.1093/jn/nxab287

**Published:** 2021-09-07

**Authors:** Kate R Schneider, Patrick Webb, Luc Christiaensen, William A Masters

**Affiliations:** Paul H. Nitze School of Advanced International Studies, Johns Hopkins University, Washington, DC, USA; Friedman School of Nutrition Science & Policy, Tufts University, Boston, MA, USA; The World Bank, Washington, DC, USA; Friedman School of Nutrition Science & Policy, Tufts University, Boston, MA, USA

**Keywords:** diet quality, household consumption and expenditure surveys, meal sharing, nutrient requirements, Malawi

## Abstract

**Background:**

Where families eat together from a common dish, the shared meal must be nutrient dense enough in each nutrient to meet the needs of the highest-need member.

**Objectives:**

This study aimed to develop an aggregate household nutrient requirement benchmark that satisfies all members’ needs in a context in which meals are shared and to illustrate how that metric could inform food and nutrition policy making.

**Methods:**

We merged nationally representative survey data for Malawi in 2010, 2013, and 2016–2017 with individual nutrient requirements and local food composition data to compute the adequacy of each household's aggregate consumption given its demographic composition and primary occupation. To meet each person's nutrient needs at any level of energy balance, the nutrient density of their shared diet needs to be within boundaries of the most restrictive member. We classified the adequacy of each household's diet using these energy-adjusted densities and examined differences by sociodemographic characteristics.

**Results:**

Accounting for meal sharing and nutrient density needs of the highest-need member, virtually all households’ food consumption is insufficiently nutrient dense in riboflavin, selenium, lipids, and vitamin B-12, and most consumption is insufficiently nutrient dense in zinc and phosphorus as well. Meeting needs of women, adolescent girls, and young children using shared diets would on average require 145% more iron, 98% more zinc, and approximately 70% more phosphorus and vitamin C than if their needs were met with individualized diets.

**Conclusions:**

Establishing shared nutrient requirements is feasible using existing survey data and can help set sufficiency criteria in settings in which families share meals. In Malawi, current diets and food composition are inadequate for many nutrients, especially in households with more women and adolescent girls. The results call for concerted investment to increase access to and use of more nutrient-dense foods.

## Introduction

Access to a high-quality diet year-round is essential for all individuals to achieve optimal growth and long-term health (1). Household consumption and expenditure surveys (HCES) offer a useful, albeit imperfect, source of information on food acquisition, especially in low-income settings with few individual dietary surveys. HCES are conducted worldwide, collected frequently, and lower cost than dietary intake surveys. National representativeness offers the potential for subnational disaggregation by geography and socioeconomic groups (2–5). Numerous studies have used them for designing food and nutrition policies and targeting interventions (6).

HCES pose the challenge for dietary assessment that they collect food consumption data at the household level, whereas nutrient requirements are individually defined. The standard approach to address this challenge is to allocate foods to individuals by apportioning dietary energy in proportion to estimated individual energy requirements (7–9). This ignores differences in nutrient density needs across individuals, such as adolescent girls needing more iron per calorie consumed than adult men. Such omission is especially problematic in informing policy when eating from shared plates is common, as has been documented throughout Asia and in low-income countries (10–15). To overcome this, we developed aggregate household nutrient requirements based on nutrient density and energy needs for a diet shared by members with heterogeneous requirements. This method has been suggested but not yet applied in practice, to the best of our knowledge. The underlying premise is that the diet must be sufficiently nutrient-dense to meet all members’ needs when each eats a share based on their individual energy requirement (16, 17). A 2012 review of existing evidence across countries concluded that the intrahousehold distribution of dietary energy can reasonably be assumed to be equitable in terms of meeting energy needs (18).

Household nutrient adequacy can then be assessed by comparing household consumption to this shared nutrient requirement. The diet is inadequate if it fails to meet the needs of ≥1 member and excessive if it exceeds the upper limit of ≥1 member. This benchmark takes account of variation in the demographic composition of each household. The importance of a more holistic household approach has also been raised in other contexts. For example, a study found that approximately half of all undernourished women and children are located outside of the poorest households (19). That study, like this one, reveals that reaching all poor and malnourished people requires attention to the whole household in which they live.

Our study offers the first practical application of the nutrient density approach to developing nutrient requirements for groups composed of members with different individual nutrient needs. Three arguments favor its use: practicality, equity, and policy relevance. There are many settings in which only household data are observed and diverse family members share meals. Shared requirements are a pragmatic approach to ask whether all members’ requirements are met. Normatively, using the shared benchmark assesses the welfare of the household by the welfare of its worst-off member, akin to Rawls’ ethical maximin principle (20, 21). Finally, adolescent girls and women of reproductive age have the highest requirements for most nutrients, making the nutrient density of shared meals an important aspect of gender equity. Shared requirements then call for the whole family to consume a diet that meets *her* minimum needs. From a policy perspective, this is a particularly useful way to evaluate households in a gender-equitable manner without observing intrahousehold behavior. Application of the method to Malawi shows how using shared requirements as a benchmark could inform policy actions to help each household acquire diets that meet all of the members’ needs.

## Methods

We used household survey data from the 3-round Malawi Integrated Household Panel Survey collected by the National Statistics Office (NSO) in 2010, 2013, and 2016–2017 (22). The data are representative at the national level and for rural and urban areas. **Table 1** provides summary statistics of the sample population. We calculated food and total expenditure and classified households into wealth quintiles using an asset index augmented with housing characteristics (23–25). Food consumption was collected by recall at the household level, asking the respondent most knowledgeable about the household's food to report the total quantity of food items (using an extensive list) consumed by all the household members during the prior 7 d (5, 26). This follows recommended practice for household consumption data collection (27).

**TABLE 1:** Population demographic characteristics and survey sample^1^

	2010	2013	2016–2017	Overall
Household size	4.88 ± 0.0355	4.88 ± 0.0888	5.102 ± 0.0816	4.97 ± 0.0774
No. of adult (>18 y) members	2.15 ± 0.0430	2.15 ± 0.0408	2.27 ± 0.0397	2.19 ± 0.0316
No. of child (≤18 y) members	3.57 ± 0.0811	3.59 ± 0.0808	3.64 ± 0.0842	3.60 ± 0.0608
N. of child members <3 y	0.914 ± 0.0267	0.785 ± 0.0280	0.74003 ± 0.0276	0.829 ± 0.0154
Dependency ratio (<15 y or >64 y defined as dependent)	1.17 ± 0.0399	1.19 ± 0.0414	1.036 ± 0.0272	1.12 ± 0.0313
Under 5 stunting,^2^ %	0.333 ± 0.0250	0.272 ± 0.0247	0.271 ± 0.0157	0.299 ± 0.0162
Rural, %	0.830 ± 0.0355	0.827 ± 0.0361	0.818 ± 0.0275	0.825 ± 0.0317
Head education, y	5.69 ± 0.228	5.79 ± 0.235	6.0031 ± 0.202	5.83 ± 0.202
Spouse education, y	3.50 ± 0.181	3.63 ± 0.205	3.61 ± 0.155	3.57 ± 0.161
Food spending share of total expenditures	0.681 ± 0.0105	0.693 ± 0.00791	0.682 ± 0.00618	0.684 ± 0.00627
Observations,^3^*n*				
Households	1615	1982	2505	6102
Individuals	7375	9534	11,540	28,449
Excluded,^4^*n*				
Individuals, no meals	141	294	503	938
Infants	147	172	215	534

1 Unless otherwise indicated, values are estimated means ± SEs for a nationally representative sample of households, estimated with sampling weights.

2 Under 5 stunting is defined as >2 SD below median, combines moderate and severe.

3 Sample size grows as all individuals from baseline households are tracked, and all members of their households are included in the subsequent data rounds should they enter new households or new members enter existing households (28). Excludes 15 households reporting that no members consumed any meals during the prior 7 d.

4 Excludes individuals eating no meals in the household in the past 7 d and all infants <6 mo, assumed to be exclusively breastfeeding.

### Defining individual nutrient needs

We categorized individuals into the 22 nonpregnant age–sex groups defined in the DRIs (29). We assumed all mothers of a child aged <2 y in the same household were breastfeeding. Pregnancy status was not collected; we applied nonpregnant nutrient needs to all women of reproductive age.

We included all micronutrients for which sufficient evidence to set an estimated average requirement (EAR) existed as of 2019 (vitamins A, B-6, B-12, C, and E; thiamin; niacin; riboflavin; folate; calcium; copper; iron; magnesium; phosphorus; selenium; and zinc), with the exception of vitamin D, iodine, and molybdenum (30, 31). For minimum nutrient requirements, we used the EAR and the lower bound of the acceptable macronutrient distribution range (AMDR). For the upper limits, we used the AMDR upper bound, upper level (UL) where it is possible to reach by consuming foods alone (i.e., not supplements) from foods, and the chronic disease risk reduction (CDRR) for sodium (29, 31). We assumed low bioavailability of iron and zinc (10% and 22% fractional absorption, respectively) (29, 32–34). We calculated individual energy requirements using WHO growth standards and reference median heights and weights specific to age, sex, and the amount of physical activity recommended for optimal health and also the most likely activity level for most Malawians (29, 35–37). We assumed a very active level for adult men as the most frequent among reported occupations, but where data were not reported for nearly half the sample, we applied an active level for the small number who reported sedentary occupations (29, 38, 39). Needs were scaled by extent of meal taking for any member who did not eat meals with the household for all 7 prior d (40).

### Defining shared household nutrient needs

To operationalize household requirements, we used the concept of nutrient density, defined as the quantity of each nutrient per unit of energy the person requires. Nutrient density with respect to energy content is often used to compare foods of varying water content. Here, we used it to compare the nutrient requirements of people with different individual energy needs. To aggregate nutrient requirements at the household level, we defined the shared nutrient density need/limit as the density of each nutrient per unit of energy meeting the highest of the minimum requirements and the lowest of the maximum upper bounds. Specifically, the maximum nutrient density (quantity per kilocalorie) required by any 1 member (aged ≥3 y) set the level for the whole household (16, 17). The household needs for each nutrient were then obtained as the shared nutrient density multiplied by total energy requirements of members aged ≥3 y plus the individual needs (from complementary foods where breastfeeding) of the younger children (41–43). The AMDR varies for protein and lipids over the life course, so we used the maximum lower and minimum upper bounds. We used the lowest UL (CDRR for sodium) in terms of nutrient density to define the household's shared upper tolerance (see **Supplemental Methods**).

### Energy-adjusted household adequacy ratios

We converted all food items consumed by the household into kilograms of edible matter using reference weights provided by the NSO and USDA, and we identified their nutrient composition using the Malawi Food Composition table supplemented by USDA data in a few cases and only where the item–nutrient was deemed unlikely to be affected by location-specific factors (44–50). Where no nutrient composition existed for a food, we assumed zero content. We discuss the implications and limitations of this assumption later.

To assess the household diet quality, we energy-adjusted the reported consumption, customarily done in epidemiological studies to address measurement error (51, 52). In low-income settings, energy-adjusting disentangles insufficient food quantity from poor diet quality, particularly where there may be measurement error. In this application, there could be recall error in the numerator (food consumed) and/or inaccurate estimates in the denominator (requirements); energy-adjusting thus focuses on the nutrient density of the diet, as opposed to overall intake adequacy (8). Household nutrient adequacy ratios compare energy-adjusted household consumption to household shared requirements (51, 52). Households were classified as having insufficient nutrient density in the diet if the ratio relative to the lower bound was <1, and they were classified as having excess nutrient density if the ratio with respect to the upper bound was >1.

### Socioeconomic analysis

We disaggregated energy-adjusted household adequacy classifications by household composition and factors typically associated with diet quality: urban/rural status and household wealth. To estimate the impact of specific types of members, we pooled the data across the 3 rounds and regressed the binary adequacy ratio on membership dummy variables of member types most often defining the shared need/limit. No covariates were included.

## Results

### Household nutrient needs

**FIGURE 1:**
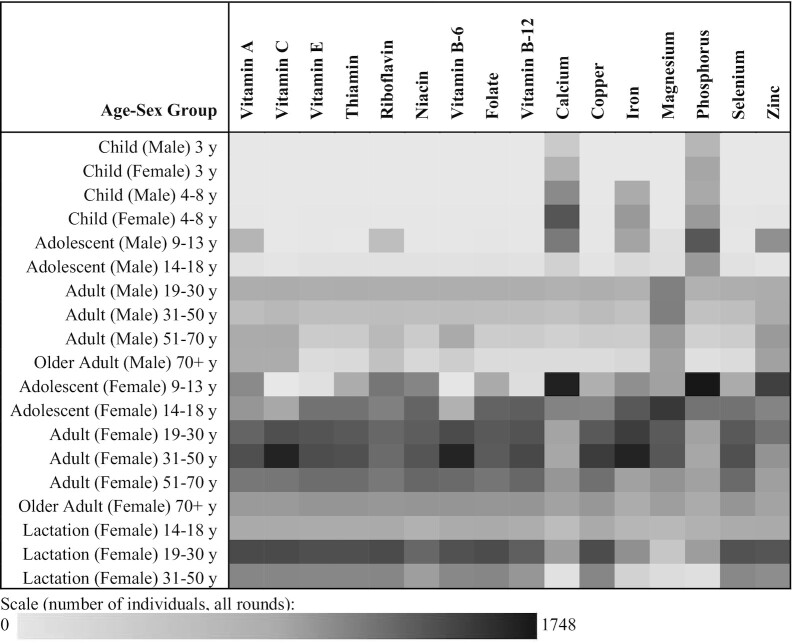
Household member with defining nutrient density need by age-sex group, per nutrient. Columns sum to the total number of individuals defining their household need. In some cases, 2 individuals have the same need and that need is defining, so the column sum is not exactly equal to the total number of household observations (6102) but it is approximately. Rows reveal large differences in the frequency with which a household member of each demographic group has the highest requirement in their household. The demographic group that is most frequently observed to define their household's needs is adolescent females aged 9–13 y, whose calcium and phosphorus requirements set their household's shared needs in approximately one-fourth of all observations. Macronutrients are not shown because many age–sex groups share the same AMDR lower bounds. AMDR, acceptable macronutrient distribution range.

**Figure 1** illustrates the frequency with which each age–sex group defined the nutrient density need for their household, reflecting differences in their nutrient density needs relative to the other demographic groups. Moving from top to bottom for each column, the darkest tile illustrates which group most frequently determined the highest need for that nutrient. For example, adolescent girls aged 9–13 y most often determined the household need for calcium (1635 instances over all 3 rounds).

Moving from left to right in Figure 1 indicates how frequently a particular age–sex group defines the household need with respect to each nutrient. For most nutrients, adolescent girls and women have higher nutrient density needs than men due to men's higher need for energy and thus lower nutrient density requirements. Where men define the household need, it indicates no other member types (not reported in Figure 1). Therefore, the diet that meets women and adolescent girl's minimum needs will meet the needs of all others as well. If no adolescent girl is present but younger children are, their needs, rather than those of an adult woman, define the household requirement for calcium and phosphorus.

**FIGURE 2:**
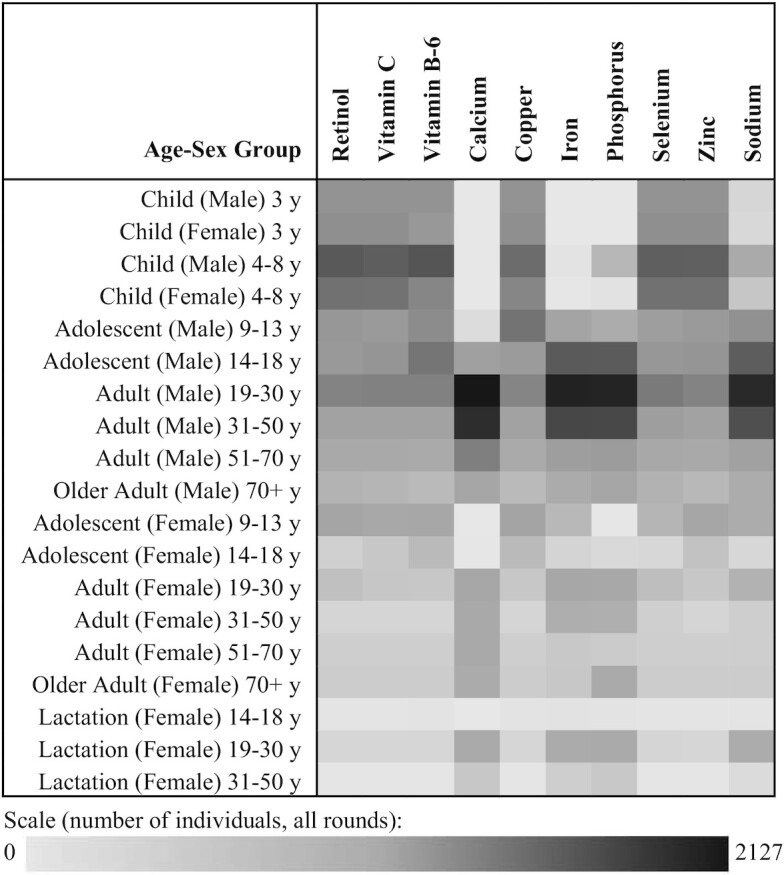
Household member with defining nutrient density tolerance by age–sex group. Data shown are the frequency at which each demographic group provides the binding upper level for each nutrient. Columns sum to the total number of individuals defining their household tolerance. In some cases, 2 individuals have the same need and that need is defining, so the column sum is not exactly equal to the total number of household observations (6102) but it is approximately. The demographic group that is most frequently observed to define their household's upper bound is adult males aged 19–30 y. Macronutrients are not shown because many age–sex groups share the same AMDR upper bounds. AMDR, acceptable macronutrient distribution range.

**Figure 2** shows which household members have the defining upper tolerance. Children and men are more likely to define the upper limits for the household compared with adult women. For instance, for calcium, adult men defined the upper tolerance in 2303 cases (37.7% of household observations). Children aged <8 y are most likely to define the upper threshold for their household for retinol, vitamin C, vitamin B-6, copper, selenium, and zinc. No households had incompatible lower and upper nutrient density bounds.

Figures 1 and 2 illustrate that meeting the needs for the whole family often requires sufficient nutrient density at the lower bound to satisfy women and girls’ needs without exceeding the limits of young children and adult men. In other words, sharing tightens the optimal range of nutrient densities compared with eating alone (or from individually adjusted plates). **Table 2** demonstrates the magnitude of this tightening, summarizing average needs (limits) at the population level under sharing relative to what it would be if the demographic composition of households were ignored. The household sharing column is the mean need (limit) for every individual at the shared diet level of nutrient density, where variation reflects the differences of household composition.

**TABLE 2:** Minimum requirements and maximum limits/1000 kcal^1^

	Shared requirements over all individuals in each household	Individual requirements ignoring household composition	% difference
Nutrient needs			
Iron, mg	6.90 ± 0.0190	3.24 ± 0.0278	145 ± 0.813
Zinc, mg	6.35 ± 0.0145	3.29 ± 0.0103	98.3 ± 0.520
Phosphorus, mg	511 ± 2.88	334 ± 1.53	69.6 ± 0.966
Vitamin C, mg	33.3 ± 0.113	22.8 ± 0.109	68.3 ± 1.24
Vitamin B-6, mg	0.621 ± 0.00285	0.447 ± 0.00206	45.1 ± 0.585
Calcium, mg	590 ± 2.01	441 ± 2.16	43.1 ± 0.564
Copper, mg	0.371 ± 0.000951	0.278 ± 0.000997	37.5 ± 0.408
Magnesium, mg	138 ± 0.465	106 ± 0.514	37.2 ± 0.513
Folate, μg	171 ± 0.396	130 ± 0.453	35.2 ± 0.379
Selenium, μg	23.3 ± 0.0542	17.8 ± 0.0663	34.8 ± 0.348
Vitamin B-12, μg	1.02 ± 0.00257	0.778 ± 0.00299	34.2 ± 0.309
Vitamin A, μg	288 ± 1.11	221 ± 0.732	33.2 ± 0.633
Vitamin E, mg	6.30 ± 0.0148	4.92 ± 0.0171	32.6 ± 0.330
Thiamin, mg	0.472 ± 0.00107	0.384 ± 0.00126	24.8 ± 0.287
Riboflavin, mg	0.488 ± 0.00106	0.400 ± 0.00128	22.8 ± 0.307
Niacin, mg	5.65 ± 0.0132	4.69 ± 0.0155	22.6 ± 0.255
Lipids,^2^ g	28.8 ± 0.0900	23.9 ± 0.0803	20.4 ± 0.317
Protein,^2^ g	24.7 ± 0.0314	22.8 ± 0.0684	10.9 ± 0.317
Carbohydrate,^2^ g	111 ± 0.145	107.6 ± 0.284	1.96 ± 0.148
Upper limits			
Copper, mg	1.77 ± 0.0235	3.10 ± 0.0199	–36.8 ± 0.437
Zinc, mg	8.45 ± 0.0770	13.3 ± 0.0682	–32.1 ± 0.291
Retinol, g	0.661 ± 0.000530	1.03 ± 0.000474	–30.8 ± 0.248
Vitamin C, mg	453 ± 3.70	683 ± 3.54	–29.7 ± 0.265
Calcium, g	912 ± 3.52	1465 ± 11.2	–27.4 ± 0.271
Selenium, μg	102 ± 0.731	146.5 ± 0.610	–27.3 ± 0.289
Iron, mg	16.5 ± 0.0646	24.8 ± 0.220	–22.6 ± 0.253
Phosphorus, g	1.42 ± 0.0483	1.94 ± 0.0831	–22.0 ± 0.230
Vitamin B-6, mg	27.0 ± 0.113	35.6 ± 0.152	–21.3 ± 0.174
Sodium,^3^ mg	800 ± 2.50	998 ± 3.57	–17.3 ± 0.196
Protein,^2^ g	66.4 ± 0.346	75.0 ± 0.222	–11.6 ± 0.350
Lipids,^2^ g	38.5 ± 0.0496	37.7 ± 0.101	0.672 ± 0.147
Carbohydrate,^2^ g	161 ± 0.206	156 ± 0.411	1.71 ± 0.147

1 Values are means ± SEs for national average population statistics using sampling weights, based on 28,449 observations of individuals and 6102 household observations. The individual column is the mean over all age–sex groups at the individual requirement level. The final column compares the 2 in percentage terms (on average). Nutrients are sorted on percentage difference, illustrated in Supplemental Figure 1. **Supplemental Table 1** presents the total quantities per day. AMDR, acceptable macronutrient distribution range; CDRR, chronic disease risk reduction.

2 Macronutrient needs and limits are defined by AMDR lower and upper bounds, respectively. Slight differences for carbohydrates are due to partial meal taking. The AMDR range does not change under household sharing because it is constant across all individual types.

3 Sodium upper bound defined by CDRR.

The optimal nutrient density intake range narrows more for iron, zinc, phosphorus, and vitamin C than for any other nutrients (see **Supplemental Figure 1**). On average, sharing would require 145% more iron, 98% more zinc, and ∼70% more phosphorus and vitamin C than if individuals ate different diets.

### Prevalence of suboptimal diet quality

**Table 3** shows the prevalence of suboptimal (inadequate or excess) nutrient density based on the dichotomous energy-adjusted adequacy ratios. More than half of all households consumed diets with inadequate nutrient density to meet minimum needs from shared diets for 13 of the 19 nutrients analyzed. **Supplemental Table 4** provides food group contributions to nutrient intakes. Virtually all had diets insufficiently dense in riboflavin, selenium, lipids, and vitamin B-12; 80% of the population consumed more calories from carbohydrates than recommended; and 50% consumed diets too dense in copper and iron, largely from plant-based sources. Supplemental Tables 2 and 3 present continuous adequacy ratios with and without energy-adjusting and for energy itself.

**TABLE 3:** Prevalence of poor diet quality, by nutrient^1^

	Nutrient density of the diet	
	Insufficient^2,3^	Excessive^3^
	% households	% households
Riboflavin	97.9 ± 0.382	—
Selenium	97.9 ± 0.352	0.749 ± 0.233
Vitamin B-12	96.0 ± 0.5031	—
Lipids	95.6 ± 0.468	1.99 ± 0.292
Zinc	86.0 ± 1.036	7.54 ± 0.647
Phosphorus	83.0 ± 0.834	0.0310 ± 0.0244
Niacin	79.3 ± 1.43	—
Vitamin E	76.5 ± 1.501	—
Vitamin A	74.8 ± 1.43	—
Calcium	71.9 ± 1.304	11.7 ± 0.902
Folate	70.3 ± 1.46	—
Protein	63.9 ± 1.34	0.413 ± 0.105
Vitamin C	49.9 ± 1.47	0.238 ± 0.125
Vitamin B-6	33.4 ± 1.67	—
Thiamin	22.0 ± 1.61	—
Carbohydrate	13.7 ± 1.013	80.8 ± 1.32
Iron	10.2 ± 0.970	51.4 ± 1.61
Magnesium	4.78 ± 0.472	—
Copper	0.743 ± 0.163	47.6 ± 1.47
Sodium^4^	—	62.4 ± 1.57
Retinol	—	0.0362 ± 0.358

1 Values are nationally representative population means ± SEs, estimated using sampling weights (*n* = 6102). Continuous adequacy ratios with and without energy-adjusting and for energy itself are presented in **Supplemental Tables 2** and **3**. AMDR, acceptable macronutrient distribution range; CDRR, chronic disease risk reduction; UL, upper limit.

2 Nutrients sorted by the estimated prevalence of inadequate nutrient density relative to the shared household requirement.

3 Dashes reflect nutrients with no UL that can be reached with food sources. Estimates of zero reflect nutrients where a UL (AMDR upper bound, CDRR) exists but the estimated prevalence of excess nutrient density in household diets is zero.

4 Nearly all sodium is from table salt, likely reflecting reporting error because it is typically purchased infrequently in larger amounts.

### Household composition and socioeconomic characteristics

Considering how household diet quality with respect to shared requirements may differ for various types of households raises 2 questions relevant to targeting programs: How does the presence of nutritionally demanding or sensitive members affect their household's classification? and How do results differ in other sociodemographic dimensions often associated with diet quality and used for targeting, namely rural/urban location and wealth?

**Table 4** presents the difference in the percentage of households classified as having suboptimal nutrient density with a member of each type relative to households without that type. These results reveal which types of households drive the national average results observed in Table 3. Table 4 shows that a greater percentage of households with adolescent girls, adult women of reproductive age, or breastfeeding women are classified as having diets insufficiently dense in vitamins A, C, and E and in niacin, calcium, iron, phosphorus, and zinc. The magnitude of difference is <10% for most individual-nutrient combinations. It is greater for calcium and zinc, meaning the population averages in Table 3 are likely influenced by households with these types of members. At the upper bound, ∼40% more households with children aged 3–8 y and ≥1 adult male are classified as having diets excessively dense in copper compared with households without children aged 3–8 y and ≥1 adult male.

**TABLE 4:** Probability of suboptimal nutrient density, by household composition^1^

	Lower bounds				Upper bounds		
	Adolescent girl (aged 9–18 y) is present	Woman of reproductive age (aged 19–50 y) is present	Breastfeeding woman is present	Constant (all other households)	Child aged 3–8 y is present	Adult male (aged 19–50 y) is present	Constant (all other households)
Carbohydrate	–6.86 ± 1.18***	–1.78 ± 2.23	1.21 ± 2.02	19.7 ± 2.22***	5.07 ± 1.89**	–5.11 ± 1.92**	79.3 ± 2.32***
Protein	–10.9 ± 1.80***	–9.19 ± 1.94***	–8.13 ± 1.99***	81.6 ± 1.90***	0.533 ± 0.171**	–0.271 ± 0.247	0.212 ± 0.159
Lipids	1.88 ± 1.00	0.913 ± 1.09	0.970 ± 0.831	93.4 ± 1.07***	–0.0867 ± 0.511	0.131 ± 0.612	2.36 ± 0.759**
Vitamin A	2.34 ± 1.37	4.10 ± 1.96*	11.2 ± 1.99***	67.1 ± 2.05***	—	—	—
Retinol	—	—	—	—	0.0360 ± 0.0351	0.0299 ± 0.0291	–0.0169 ± 0.0165
Vitamin C	5.85 ± 1.66***	–0.498 ± 2.07	15.9 ± 2.59***	41.9 ± 2.32***	0.187 ± 0.205	–0.00865 ± 0.178	0.152 ± 0.153
Vitamin E	10.9 ± 1.53***	–1.37 ± 1.89	4.91 ± 2.17*	68.3 ± 2.28***	—	—	—
Thiamin	2.42 ± 1.52	–0.350 ± 2.04	1.38 ± 2.19	20.0 ± 1.95***	—	—	—
Niacin	1.25 ± 0.589*	–0.402 ± 1.08	1.56 ± 0.871	96.6 ± 0.916***	—	—	—
Riboflavin	7.94 ± 1.58***	2.36 ± 1.91	–2.99 ± 2.49	73.2 ± 1.96***	—	—	—
Vitamin B-6	3.12 ± 1.99	–6.57 ± 2.35**	2.43 ± 2.49	34.2 ± 2.58***	—	—	—
Folate	2.48 ± 1.53	4.35 ± 2.07*	14.6 ± 2.12***	61.2 ± 2.41***	—	—	—
Vitamin B-12	1.40 ± 0.884	–1.79 ± 1.08	–0.454 ± 0.996	95.9 ± 0.789***	—	—	—
Calcium	13.2 ± 1.73***	4.58 ± 1.93*	4.21 ± 2.41	58.8 ± 2.07***	–3.45 ± 1.09**	–0.287 ± 1.10	14.4 ± 1.26***
Copper	–0.203 ± 0.321	–0.0318 ± 0.550	–0.626 ± 0.478	1.06 ± 0.489*	31.9 ± 1.79***	7.78 ± 1.86***	17.3 ± 1.84***
Iron	2.55 ± (1.07)*	5.80 ± (1.40)***	1.30 ± (1.34)	4.86 ± (1.18)***	1.10 ± (1.75)	7.31 ± (2.97)*	44.2 ± (2.55)***
Magnesium	1.27 ± 0.761	–0.697 ± 1.08	–2.58 ± 1.16*	5.32 ± 1.10***	—	—	—
Phosphorus	34.5 ± 1.44***	7.03 ± 1.81***	1.50 ± 1.67	56.4 ± 1.89***	–0.150 ± 0.107	–0.0560 ± 0.118	0.195 ± 0.167
Selenium	0.355 ± 0.507	–0.840 ± 0.651	0.476 ± 0.577	97.9 ± 0.610***	–0.282 ± 0.342	0.939 ± 0.293**	0.328 ± 0.168
Zinc	13.0 ± 1.55***	–3.23 ± 1.67	12.3 ± 1.62***	76.4 ± 2.01***	8.40 ± 1.05***	0.335 ± 1.01	1.51 ± 0.603*

1 Values are the difference in probability that the nutrient indicated by each row label will be inadequate when the household includes a member of the demographic group (columns) ± SEs, computed over *n* = 6102 household observations, nationally representative using sampling weights. ^*,**,***^Significance levels are denoted as follows: *P < 0.05, **P < 0.01, ***P < 0.001.

**FIGURE 3:**
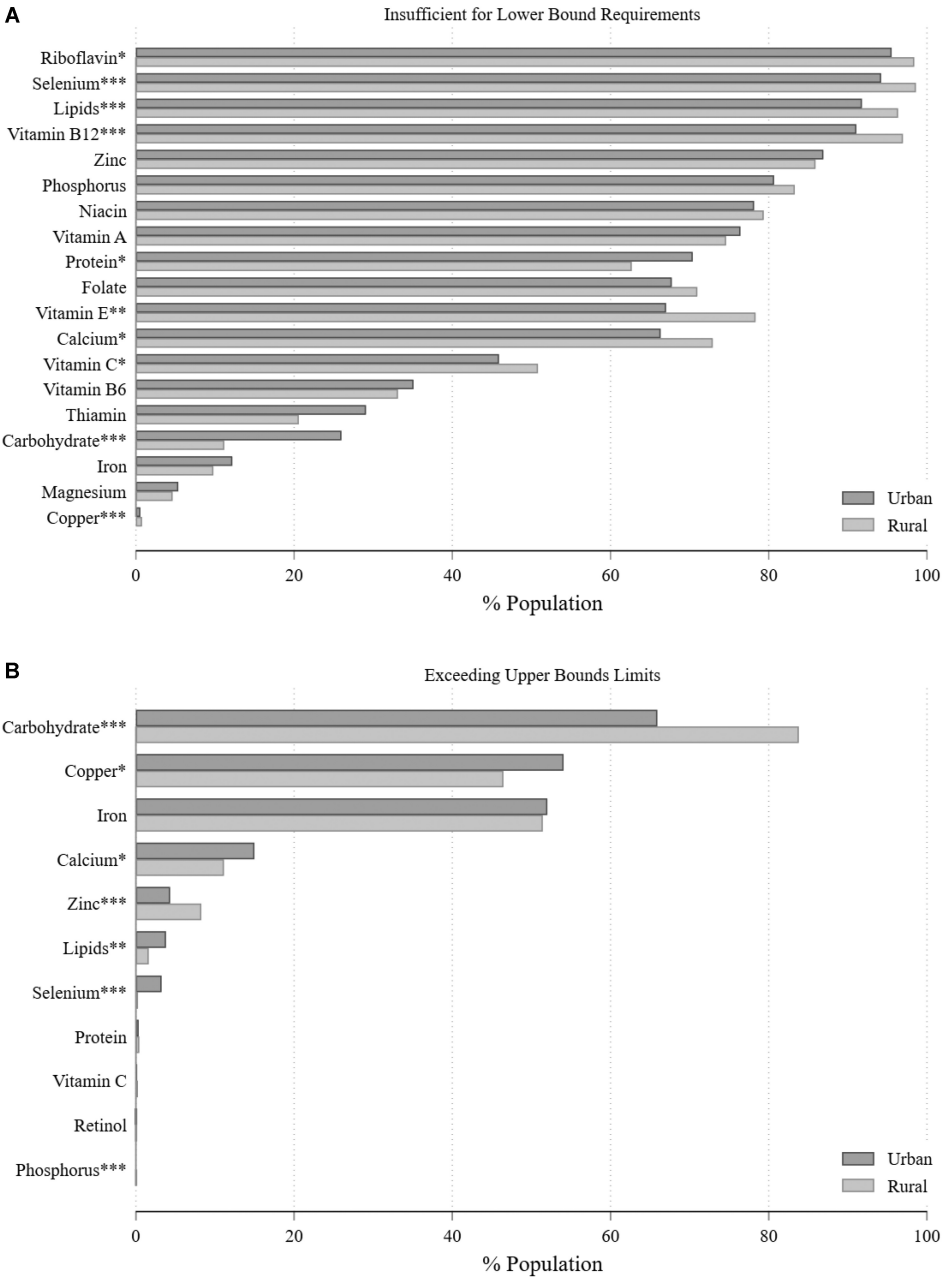
Prevalence of suboptimal nutrient density in Malawian households, by urban/rural status. Population statistics corrected using sampling weights. ^*,**,***^Statistically significant difference by urban/rural status: *P < 0.05, **P < 0.01, ***P < 0.001.

**FIGURE 4:**
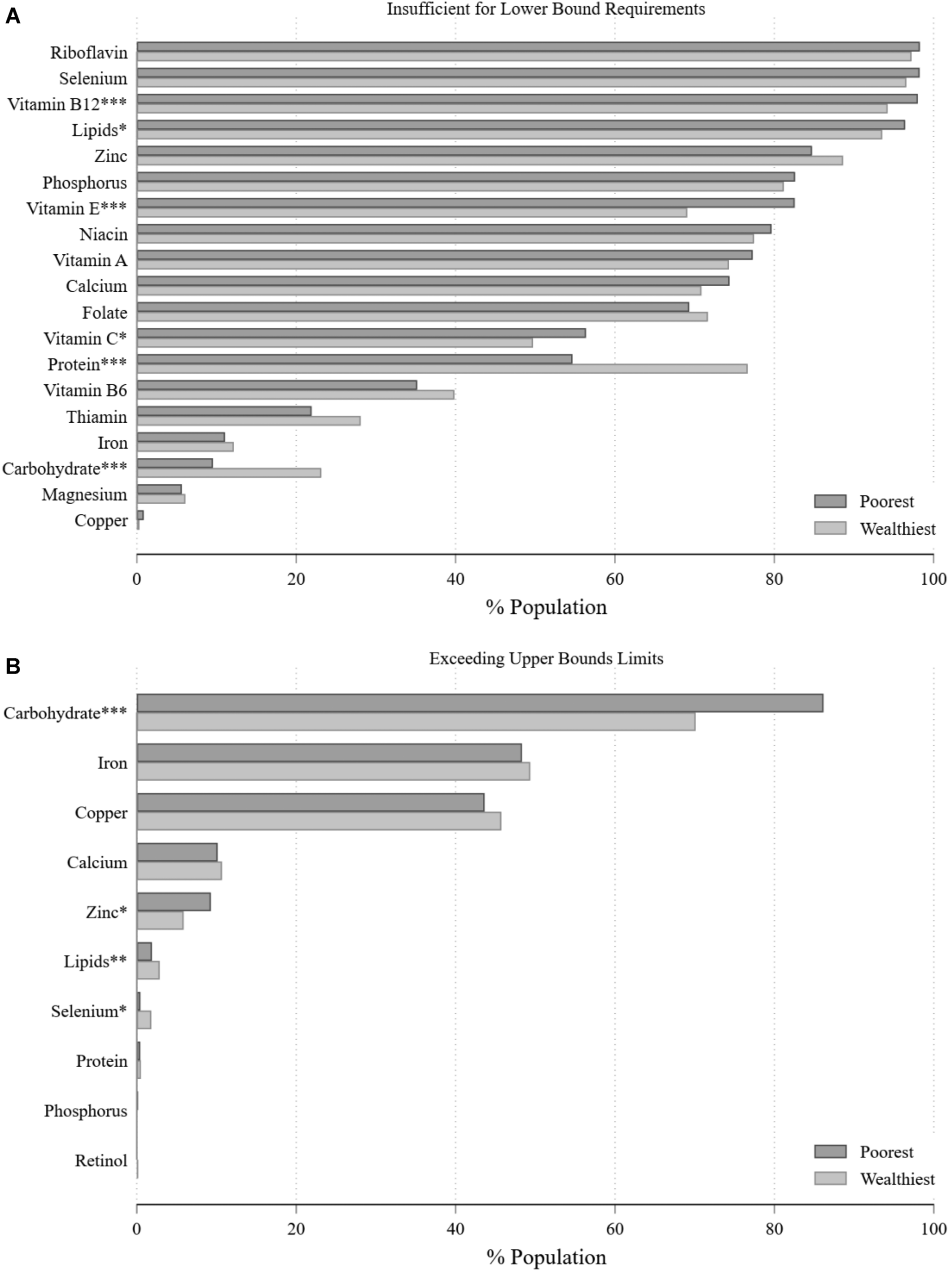
Prevalence of suboptimal nutrient density in Malawian households, by wealth quintile. Population statistics corrected using sampling weights. ^*,**,***^Statistically significant difference between the wealthiest and poorest quintile: *P < 0.05, **P < 0.01, ***P < 0.001.

**Figures 3** and **4** disaggregate the results by 2 socioeconomic characteristics commonly associated with diet quality: rural/urban location and household wealth. These figures show that rural and the poorest (lowest quintile) households are, as would be expected, more likely to be classified as having insufficient nutrient density in the diet relative to urban and the wealthiest (highest quintile) households for most nutrients. However, differences are generally small even where statistically significant. Overall, the difference between Malawi's rural and urban populations is less than one might expect, reflecting the country's very low household income and continuing reliance on maize-based diet even in its cities and the fact that the vast majority of the country's population remains rural (83% as of 2019) (53).

## Discussion

We developed household-level nutrient requirements using a shared diet adequate in all nutrients for all members of a household. We defined the shared requirement per nutrient by the nutrient density need/limit of the member with the most restrictive requirement (highest lower bound need or most restrictive upper tolerance). We estimated this shared nutrient requirement for a nationally representative sample of households in Malawi and illustrated how it classifies the nutrient adequacy of current diets.

We found most households in Malawi consumed diets with inadequate nutrient density to meet minimum needs of all members from shared diets. The nutrients of concern at the lower bound were riboflavin, selenium, lipids, vitamin B-12, zinc, and phosphorus. Adolescent girls, women of reproductive age, and breastfeeding mothers most often had the defining needs for these nutrients, but diets were insufficiently dense in these nutrients to meet their needs. Households with such members underpinned the insufficiency of vitamin C and zinc in many diets.

At the upper bound, most households (80%) consumed more calories from carbohydrates than recommended. Even among the wealthiest households, 60% still exceeded the recommended percentage energy from carbohydrates. This reflects Malawi's dietary and policy focus on maize. A large share of households also consumed a diet too dense in copper and iron, particularly in households with children aged 3–8 y and adult men together. Local food composition data reveal relations between soils and diets. The vast majority of iron and copper came from plant-based sources (Supplemental Table 4), reflecting uptake from Malawi's soils, which are rich in iron and copper. The reverse is true for selenium, where widespread soil deficiency is apparent in inadequate selenium intake (50, 54). Primary data collection could further validate these apparent soil-related findings. Monitoring a sample of the population for copper and iron toxicity can determine whether there is cause for concern. Because we found few differences by location or wealth, given current diets, this shared nutrient requirement benchmark may be too demanding to meaningfully classify households to target interventions. There also may not be enough nutrient-dense foods available, or they may not be affordable, to provide for the high level of diet quality sharing food would require. This is an important finding in Malawi and relevant to all places where shared plate eating is the cultural norm. Both making adequate shared diets financially affordable and influencing household behavior to target nutrient-dense foods to the neediest members could improve diet quality in Malawi.

Our finding that there was little variation across the population suggests the most promising interventions will increase the availability and access to nutrient-dense foods throughout the country, especially foods that are good sources of riboflavin, selenium, lipids, vitamin B-12, zinc, and phosphorus. Animal-source foods (ASFs) are rich in all such nutrients, and therefore investments in livestock production and productivity, transport, and cold storage could reduce the current barriers to accessing ASFs: increasing availability and reducing costs to consumers. At current incomes and food prices, increasing access to high-quality diets requires increasing incomes (or transfers), reducing prices, or both. In related work, we estimated the cost of the shared diet and tested policy scenarios to increase availability and reduce cost (55). The results could also be used to develop nutrition education and behavior change materials to encourage food targeting within households, thereby alleviating the need for all members to eat the high-quality diet sharing food demands. Such actions would be useful even where income, production, and productivity investments are made, given the time lag between program implementation and outcomes.

A number of assumptions were inevitably made in arriving at our conclusions due to data limitations, although they are unlikely to affect the results. Particularly, in estimating nutrient requirements, we assumed breastfeeding for all women with children aged <2 y and did not account for pregnancy. Yet, median breastfeeding duration in Malawi is 23 mo (42), and erroneous assumptions would only classify a household as having insufficient nutrient density for the nutrients defined by the breastfeeding woman due to lactation (i.e., vitamin A). Excluding pregnant women also minimally affects our results because they only define the need for folate and iron, and these needs are best met through prenatal vitamins (56–58). Furthermore, in calculating nutrient intakes, food consumed away from home and any nutrient supplementation were also ignored. Urban, wealthier, and larger households are more likely to eat food away from home and to have adult members consume food outside of the visibility of the reporting individual (59–62). Only 2.3% of all households reported a restaurant meal (1.6% for rural households), offering the best confirmation that food away from home is likely a small share of household food consumption. Finally, we account for industrial fortification in cooking oil and sugar but do not account for supplementation (e.g., widespread vitamin A supplementation for children aged <5 y), which would reduce the amount needed from foods (63).

In this article, we demonstrated how estimating shared nutrient requirements for whole households can assess diet quality in the context of shared plate eating. Several arguments favor using this shared benchmark, including practicality, where only household food consumption is observed and meal sharing is prevalent; equity in guiding interventions that affect whole households using the well-established normative welfare principle; and sensitivity to gender in terms of the particular needs of women and girls when evaluating household diet quality. In the Malawian context, we found few households consumed diets high enough in nutrient density to meet the needs of all their members. Our findings call for increasing use of policies, programs, and nutrition assistance to bring adequate diets within reach and for guiding households to target nutrient-dense foods to their nutritionally neediest members, namely women and girls.

## Supplementary Material

nxab287_Supplemental_File

## Notes

This work was funded by UKAid and the Bill & Melinda Gates Foundation through Changing Access to Nutritious Diets in Africa and South Asia project grant OPP1182628. PW was supported by US Agency for International Development award AID-OAA-LA-14-00012 to the Friedman School of Nutrition Science and Policy at Tufts University through the Feed the Future Innovation Lab for Nutrition.

Author disclosures: The authors report no conflicts of interest.

Supplemental Methods, Supplemental Figure 1, and Supplemental Tables 1–4 are available from the “Supplementary data” link in the online posting of the article and from the same link in the online table of contents at https://academic.oup.com/jn.

Abbreviations used: AMDR, acceptable macronutrient distribution range; ASF, animal-source food; CDRR, chronic disease risk reduction; EAR, estimated average requirement; HCES, household consumption and expenditure surveys; NSO, National Statistics Office; UL, upper level.
