# Supplementary material for: Assessing Diet Quality Where Families Share Their Meals: Evidence from Malawi
Source: J Nutr. 2021 Sep 7;151(12):3820–30. doi: 10.1093/jn/nxab287 (PMC8643595; doi:10.1093/jn/nxab287)
Supplement: nxab287_Supplemental_File [file nxab287_supplemental_file.docx]

**Assessing diet quality where families share their meals: Evidence from Malawi**

Kate R. Schneider, PhD (corresponding author)

**Online Supplemental Materials**

**Supplemental Methods: Methodology to aggregate individual nutrient requirements to the household level**

The method used to aggregate individual nutrient requirements to the household level based on nutrient density follows Beaton (1995) (subsequently described in (17)) as the appropriate method to define the diet quality required by a group of heterogeneous individuals (16). Formally, we define the shared household nutrient requirements by the individual needs of each household’s members (m) for density of each nutrient (j), multiplied by the sum of all energy requirements (e/E), using the most restrictive of their nutrient density requirements for each upper and lower bound:

${HHLower}_{j}= \sum_{m} {E_{m}*{max}_{m}\{MinimumNeed}_{j,m}/E_{m}\}, j=1,\ldots, 19$ (1)

${HHUpper}_{j} = \sum_{m} {E_{m}*{min}_{m}\{MaximumTolerance}_{j,m}/E_{m}\}, j=1,\ldots, 13$ (2)

${HHE}_{e}= \sum_{m} E_{m}$ (3)

We term the household bounds as HHE for energy, HHLower for the minimum needed amount, and HHUpper for the maximally tolerable amount, to distinguish them clearly from the energy balance, minimum needs and maximum tolerances that have been defined based on biomedical evidence for individuals.

We include lower bounds for 19 nutrients (three macronutrients and 16 micronutrients), defined for each individual by the EAR and AMDR lower bound. For 13 nutrients, we include upper bounds (three macronutrients and ten micronutrients), defined by their UL, CDRR, and AMDR upper bound. AMDRs are defined as range of percent of calories from each macronutrient. For carbohydrates, this range is identical for all individuals. For protein and lipids, the range varies slightly by age and sex group and therefore the household-level range takes the narrowest range to satisfy all individuals’ needs (highest of the lower bound and lowest of the higher bound of all individuals present in the household).

We do not use the needs of children under three years to define the shared household diet nor consider those children to eat the shared diet, their unique needs are met separately. Specifically, we calculate the household total nutrients for all other members and then add to that the needs of any children six months through two years old. Children through two years have very unique nutrient needs and are often fed separate foods; following Beaton (1995), this age group should not be included in establishing the nutrient density requirement for a heterogeneous group (16).

**Supplemental Figure 1**  Change in Optimal Nutrient Density Intake Range with Household Sharing Relative to Individual Requirements


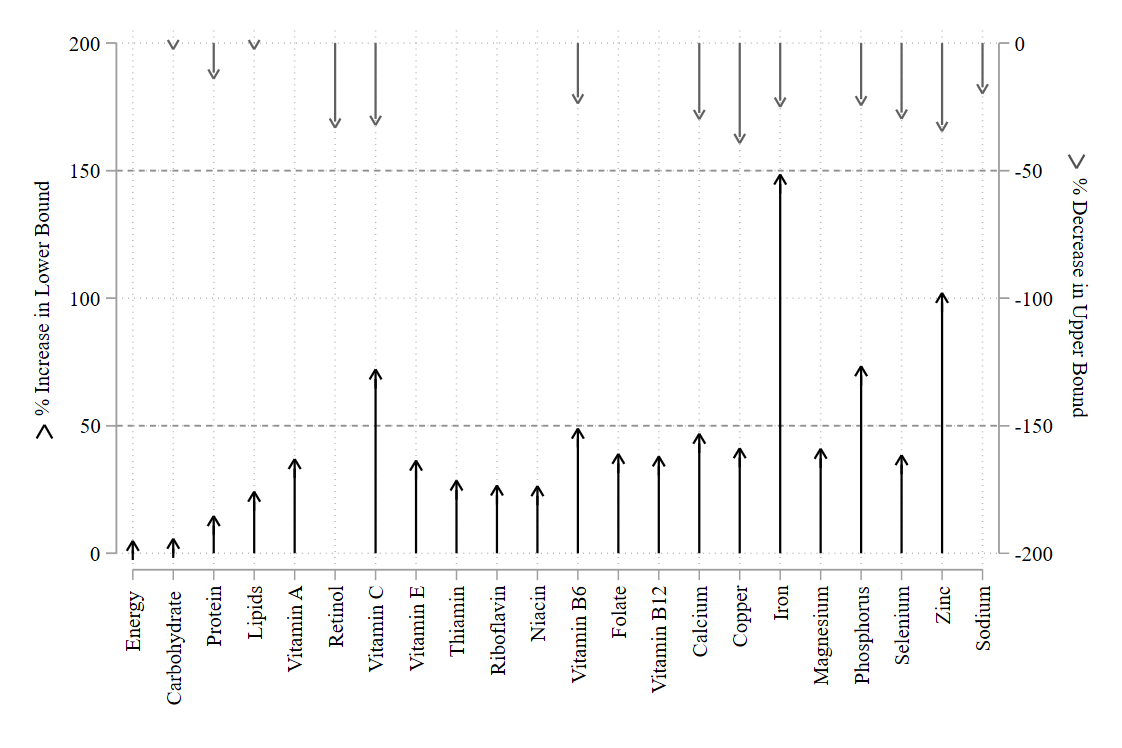


**Supplemental Table1** Nutrient Requirements and Limits Per Day, by Nutrient, in Malawian Population^1^

|  | **Nutrient Needs** | | | | | | | |  | **Limits** | | | | | | | |
| --- | --- | --- | --- | --- | --- | --- | --- | --- | --- | --- | --- | --- | --- | --- | --- | --- | --- |
|  | Individual^2^ | |  | Shared Per Capita | |  | Shared Household Total | |  | Individual^2^ | |  | Shared Per Capita | |  | Shared Household Total | |
|  | Mean | (SE) |  | Mean | (SE) |  | Mean | (SE) |  | Mean | (SE) |  | Mean | (SE) |  | Mean | (SE) |
| Energy (kcal) | 1,903 | (221) |  | 1,881 | (241) |  | 10,959 | (57.6) |  |  |  |  |  |  |  |  |  |
| Carbohydrate* (g) | 217 | (242) |  | 213 | (249) |  | 1,242 | (57.7) |  | 313 | (242) |  | 307 | (247) |  | 1,790 | (57.7) |
| Protein* (g) | 47.1 | (217) |  | 47.2 | (245) |  | 275 | (57.7) |  | 155 | (202) |  | 128 | (129) |  | 731 | (59.1) |
| Lipids* (g) | 46.9 | (278) |  | 54.7 | (272) |  | 325 | (54.6) |  | 75.4 | (249) |  | 73.6 | (248) |  | 429 | (58.0) |
| Vitamin A (μg) | 453 | (229) |  | 546 | (250) |  | 3,230 | (51.9) |  |  |  |  |  |  |  |  |  |
| Retinol (μg) |  |  |  |  |  |  |  |  |  | 2,061 | (140) |  | 1,281 | (92.6) |  | 7,043 | (62.5) |
| Vitamin C (mg) | 48.4 | (168) |  | 63.4 | (243) |  | 374.1 | (52.7) |  | 1,399 | (154) |  | 879 | (92.3) |  | 4,855 | (61.6) |
| Vitamin E (mg) | 9.60 | (234) |  | 12.0 | (228) |  | 70.7 | (55.2) |  |  |  |  |  |  |  |  |  |
| Thiamin (mg) | 0.758 | (233) |  | 0.902 | (228) |  | 5.29 | (55.3) |  |  |  |  |  |  |  |  |  |
| Riboflavin (mg) | 0.802 | (228) |  | 0.932 | (248) |  | 5.48 | (54.9) |  |  |  |  |  |  |  |  |  |
| Niacin (mg) | 9.19 | (230) |  | 10.8 | (211) |  | 63.4 | (55.0) |  |  |  |  |  |  |  |  |  |
| Vitamin B-6 (mg) | 0.890 | (185) |  | 1.18 | (175) |  | 6.97 | (51.2) |  | 70.9 | (175) |  | 51.7 | (153) |  | 292 | (62.6) |
| Folate (μg) | 258 | (229) |  | 326 | (237) |  | 1,918 | (54.8) |  |  |  |  |  |  |  |  |  |
| Vitamin B-12 (μg) | 1.56 | (210) |  | 1.95 | (202) |  | 11.4 | (54.8) |  |  |  |  |  |  |  |  |  |
| Calcium (mg) | 827 | (249) |  | 1,124 | (216) |  | 6,736 | (51.9) |  | 2,470 | (356) |  | 1,731 | (295) |  | 9,967 | (62.6) |
| Copper (mg) | 0.557 | (225) |  | 0.709 | (234) |  | 4.17 | (54.6) |  | 6.51 | (135) |  | 3.46 | (64.4) |  | 18.5 | (56.4) |
| Iron (mg) | 5.61 | (257) |  | 13.2 | (193) |  | 78.3 | (53.7) |  | 41.0 | (381) |  | 31.2 | (264) |  | 179 | (65.2) |
| Magnesium (mg) | 220 | (155) |  | 264 | (169) |  | 1,559 | (52.4) |  |  |  |  |  |  |  |  |  |
| Phosphorus (mg) | 635 | (213) |  | 976 | (147) |  | 6,009 | (46.8) |  | 3,550 | (361) |  | 2,697 | (251) |  | 15,507 | (64.3) |
| Selenium (μg) | 35.8 | (222) |  | 44.6 | (220) |  | 262 | (55.1) |  | 292 | (156) |  | 197 | (101) |  | 1,100 | (62.1) |
| Zinc (mg) | 6.44 | (207) |  | 12.1 | (252) |  | 71.6 | (54.1) |  | 27.0 | (137) |  | 16.4 | (84.9) |  | 89.8 | (61.8) |
| Sodium (mg) |  |  |  |  |  |  |  |  |  | 1,891 | (266) |  | 1,523 | (230) |  | 8,757 | (62.9) |
| **Observations** |  |  |  |  |  |  |  |  |  |  |  |  |  |  |  |  |  |
| Individuals | 28,449 |  |  |  |  |  |  |  |  |  |  |  |  |  |  |  |  |
| Households | 6,102 |  |  |  |  |  |  |  |  |  |  |  |  |  |  |  |  |

^1^ Population statistics calculated using sampling weights.

^2^ As a population-weighted average over the whole population, the individual mean values reflect both the differences in requirements by age-sex group as well as the demographic structure of the Malawian population.

**Supplemental Table 2** Mean Nutrient Adequacy Ratios by Nutrient, With and Without Energy-adjusting^1,2^

|  |  | **Reported Intakes** | |  | **Energy-Adjusted Intakes** | |
| --- | --- | --- | --- | --- | --- | --- |
|  |  | Mean | (SE) |  | Mean | (SE) |
| Energy |  | 2.00 | (0.114) |  |  |  |
| Carbohydrate |  | 2.08 | (0.127) |  | 1.16 | (0.00654) |
| Protein |  | 1.17 | (0.0688) |  | 0.822 | (0.00968) |
| Lipids |  | 1.44 | (0.133) |  | 0.601 | (0.00746) |
| Vitamin A |  | 1.48 | (0.0817) |  | 0.883 | (0.0419) |
| Vitamin C |  | 2.21 | (0.107) |  | 1.48 | (0.0493) |
| Vitamin E |  | 2.34 | (0.343) |  | 0.862 | (0.0214) |
| Thiamin |  | 3.02 | (0.343) |  | 1.67 | (0.0307) |
| Riboflavin |  | 1.18 | (0.135) |  | 0.59 | (0.0101) |
| Niacin |  | 1.63 | (0.132) |  | 0.850 | (0.0140) |
| Vitamin B-6 |  | 2.30 | (0.183) |  | 1.29 | (0.0226) |
| Folate |  | 2.03 | (0.601) |  | 0.893 | (0.0173) |
| Vitamin B-12 |  | 0.576 | (0.0682) |  | 0.253 | (0.0167) |
| Calcium |  | 1.64 | (0.217) |  | 1.01 | (0.0806) |
| Copper |  | 8.40 | (0.769) |  | 5.00 | (0.135) |
| Iron |  | 6.03 | (0.987) |  | 3.66 | (0.367) |
| Magnesium |  | 4.36 | (0.396) |  | 2.58 | (0.0754) |
| Phosphorus |  | 1.81 | (0.355) |  | 0.754 | (0.0133) |
| Selenium |  | 0.936 | (0.117) |  | 0.314 | (0.0163) |
| Zinc |  | 1.40 | (0.139) |  | 0.782 | (0.0158) |
| Observations |  | 6,102 |  |  |  |  |

^1^ This table presents continuous adequacy ratios with and without energy-adjusting underlying the prevalence of suboptimal diet quality shown in Table 3 of the main paper. Adequacy ratios are reported intake relative to estimated household shared need; energy-adjusting attempts to address recall and measurement error in reported intakes.

^2^ Population statistics calculated using sampling weights.

**Supplemental Table 3** Energy Adequacy Ratios^1,2^

|  |  | Nutrient  Adequacy  Ratio |  | (SE) |
| --- | --- | --- | --- | --- |
| Percentiles |  |  |  |  |
| 5 |  | 0.517 |  | 0.0179 |
| 10 |  | 0.642 |  | 0.0176 |
| 25 |  | 0.883 |  | 0.0176 |
| 50 |  | 1.26 |  | 0.0272 |
| 75 |  | 1.84 |  | 0.0436 |
| 90 |  | 2.78 |  | 0.106 |
| 95 |  | 3.39 |  | 0.205 |
| Observations |  | 6,102 |  |  |

^1^ Adequacy ratios are the energy intake relative to estimated energy requirement. Percentiles describe the distribution of the difference between reported energy consumption and the estimated need in the population. We estimated 40% reported consuming less energy than estimated to be required (energy adequacy ratio less than 1), while 20%.

^2^ Population statistics calculated using sampling weights.

**Supplemental Table 4** Food group contributions to nutrient totals (mean percent total nutrient intake from the food group in Malawian households)

|  | Carbohydrate | Protein | Lipids | Vit A | Retinol | Vit C | Vit E | Thiamin | Riboflavin | Niacin |
| --- | --- | --- | --- | --- | --- | --- | --- | --- | --- | --- |
| Caloric beverages | 1.27 | 0.0339 | 0.0365 | 0.000422 | 0.00100 | 2.960 | 0.0270 | 0.0544 | 0.326 | 0.102 |
|  | (0.232) | (0.0111) | (0.0118) | (0.000137) | (0.000350) | (0.638) | (0.00743) | (0.0178) | (0.0844) | (0.0348) |
| Cereals, cereal products | 31.9 | 27.6 | 21.9 | 0.205 | 33.52 | 1.18 | 17.1 | 29.0 | 22.0 | 21.2 |
|  | (0.827) | (0.777) | (0.841) | (0.0435) | (9.68) | (0.164) | (0.631) | (0.791) | (0.706) | (0.719) |
| Dark green leafy vegetables | 0.442 | 0.822 | 0.260 | 31.6 |  | 15.2 | 7.85 | 1.14 | 4.04 | 1.62 |
|  | (0.0236) | (0.0416) | (0.0245) | (1.36) |  | (0.737) | (0.379) | (0.0626) | (0.194) | (0.0788) |
| Eggs | 0.0380 | 2.22 | 2.81 | 4.33 | 57.0 |  |  | 1.13 | 7.31 | 0.121 |
|  | (0.00155) | (0.0811) | (0.0833) | (0.252) | (1.49) |  |  | (0.0472) | (0.195) | (0.00431) |
| Fish & seafood | 0.0120 | 5.82 | 6.76 | 1.03 |  |  | 0.802 | 1.03 | 6.29 | 5.65 |
|  | (0.00113) | (0.363) | (0.356) | (0.117) |  |  | (0.0715) | (0.0936) | (0.530) | (0.508) |
| Flesh meat | 0.000611 | 8.63 | 5.92 | 0.713 | 58.6 | 0.381 | 0.788 | 4.07 | 8.14 | 13.4 |
|  | (0.000146) | (0.260) | (0.250) | (0.0508) | (2.21) | (0.0421) | (0.0549) | (0.176) | (0.264) | (0.376) |
| Legumes | 2.94 | 8.03 | 7.73 | 0.397 |  | 0.521 | 7.40 | 9.84 | 6.57 | 10.6 |
|  | (0.108) | (0.209) | (0.399) | (0.0555) |  | (0.0417) | (0.423) | (0.266) | (0.185) | (0.335) |
| Milk, milk products | 0.736 | 2.17 | 3.69 | 7.08 | 36.2 | 0.811 | 0.486 | 1.01 | 10.1 | 0.538 |
|  | (0.128) | (0.206) | (0.265) | (0.729) | (2.09) | (0.102) | (0.0777) | (0.129) | (0.426) | (0.121) |
| Nuts seeds | 0.408 | 1.56 | 20.7 |  |  | 0.165 | 0.730 | 6.93 | 2.99 | 2.38 |
|  | (0.0557) | (0.0940) | (1.89) |  |  | (0.101) | (0.0762) | (1.69) | (0.642) | (0.381) |
| Oils fats | 0.00395 | 0.0128 | 20.9 | 18.6 | 37.4 |  | 31.1 | 6.17 | 13.1 | 9.000 |
|  | (0.00282) | (0.00335) | (0.609) | (4.77) | (4.14) |  | (0.865) | (0.522) | (1.16) | (0.655) |
| Other fruit | 2.33 | 0.864 | 2.43 | 2.39 |  | 13.2 | 2.47 | 1.45 | 3.49 | 3.47 |
|  | (0.128) | (0.0510) | (0.469) | (0.272) |  | (0.922) | (0.221) | (0.0950) | (0.214) | (0.247) |
| Other vegetable | 0.502 | 0.510 | 0.144 | 5.12 |  | 7.41 | 1.22 | 0.793 | 1.30 | 1.54 |
|  | (0.0133) | (0.0147) | (0.00723) | (0.253) |  | (0.266) | (0.0458) | (0.0233) | (0.0400) | (0.0455) |
| Roots tubers | 7.87 | 2.45 | 1.15 | 1.48 |  | 22.6 | 1.84 | 5.62 | 6.97 | 7.43 |
|  | (0.673) | (0.198) | (0.289) | (0.176) |  | (0.824) | (0.152) | (0.402) | (0.628) | (0.555) |
| Salty snacks & fried foods | 0.781 | 0.877 | 2.02 | 1.31 |  | 0.742 | 2.22 | 0.864 | 1.50 | 1.00 |
|  | (0.0370) | (0.0385) | (0.101) | (0.119) |  | (0.0746) | (0.141) | (0.0487) | (0.0565) | (0.0617) |
| Sweets, confectionary | 5.62 | 1.16 | 1.42 | 2.08 | 46.3 | 6.34 | 0.0954 | 2.76 | 5.75 | 1.81 |
|  | (0.162) | (0.0910) | (0.132) | (0.169) | (3.28) | (0.436) | (0.00534) | (0.274) | (0.415) | (0.208) |
| Vitamin A-rich fruits | 2.48 | 0.632 | 0.549 | 15.1 |  | 28.9 | 5.12 | 2.20 | 2.76 | 1.66 |
|  | (0.186) | (0.0441) | (0.0419) | (1.49) |  | (1.46) | (0.500) | (0.159) | (0.253) | (0.130) |
| Vitamin A-rich vegetables tubers | 7.97 | 6.37 | 1.16 | 72.7 |  | 31.1 | 18.9 | 7.87 | 12.6 | 14.5 |
|  | (0.466) | (0.468) | (0.145) | (1.04) |  | (1.17) | (1.60) | (0.686) | (0.675) | (0.742) |

|  | Vit B-6 | Folate | Vit B-12 | Calcium | Copper | Iron | Magnesium | Phosphorus | Selenium | Zinc | Sodium |
| --- | --- | --- | --- | --- | --- | --- | --- | --- | --- | --- | --- |
| Caloric beverages | 0.157 | 0.00384 | 0.0232 | 0.364 | 0.236 | 0.126 | 0.211 | 0.237 | 0.0219 | 0.441 | 0.388 |
|  | (0.0470) | (0.00147) | (0.00775) | (0.112) | (0.0972) | (0.0519) | (0.0611) | (0.0611) | (0.00724) | (0.150) | (0.185) |
| Cereals, cereal products | 21.6 | 11.8 | 0.139 | 3.46 | 11.3 | 12.3 | 18.4 | 17.8 | 41.1 | 20.3 | 3.14 |
|  | (0.732) | (0.407) | (0.0614) | (0.158) | (0.368) | (0.473) | (0.494) | (0.485) | (2.18) | (0.584) | (0.170) |
| Dark green leafy vegetables | 3.42 | 10.4 |  | 19.3 | 4.60 | 18.2 | 9.12 | 2.49 | 12.6 | 4.42 | 0.844 |
|  | (0.181) | (0.476) |  | (0.852) | (0.241) | (0.887) | (0.469) | (0.132) | (0.671) | (0.235) | (0.0742) |
| Eggs | 0.309 | 1.95 | 45.8 | 0.562 |  | 0.660 | 0.167 | 3.43 |  | 1.33 | 1.04 |
|  | (0.0122) | (0.0783) | (1.38) | (0.0251) |  | (0.0309) | (0.00825) | (0.138) |  | (0.0437) | (0.0753) |
| Fish seafood | 4.72 | 0.403 | 54.3 | 19.4 | 0.958 | 13.3 | 6.13 | 2.47 |  | 3.52 | 2.82 |
|  | (0.433) | (0.0477) | (3.71) | (0.821) | (0.137) | (0.848) | (0.373) | (0.232) |  | (0.350) | (0.430) |
| Flesh meat | 6.28 | 0.485 | 40.4 | 0.365 | 1.02 | 2.45 | 1.19 | 7.19 | 11.6 | 7.27 | 1.21 |
|  | (0.198) | (0.0406) | (1.40) | (0.0290) | (0.0698) | (0.138) | (0.0628) | (0.253) | (0.862) | (0.238) | (0.0863) |
| Legumes | 6.76 | 17.9 |  | 3.23 | 5.74 | 4.03 | 6.47 | 13.3 | 16.9 | 6.36 | 0.376 |
|  | (0.213) | (0.393) |  | (0.103) | (0.161) | (0.121) | (0.173) | (0.411) | (0.458) | (0.169) | (0.0345) |
| Milk, milk products | 1.35 | 0.997 | 23.7 | 5.65 | 0.154 | 0.588 | 1.02 | 5.26 | 1.96 | 2.00 | 1.36 |
|  | (0.157) | (0.141) | (1.47) | (0.285) | (0.0140) | (0.0784) | (0.169) | (0.315) | (0.199) | (0.201) | (0.150) |
| Nuts seeds | 1.72 | 0.429 |  | 2.63 | 3.26 | 1.82 | 2.35 | 2.31 | 6.62 | 1.56 | 0.0138 |
|  | (0.498) | (0.0758) |  | (0.276) | (0.262) | (0.251) | (0.430) | (0.588) | (0.621) | (0.386) | (0.00284) |
| Oils fats | 7.48 | 4.57 | 7.98 | 0.154 | 0.0346 | 0.000858 | 0.0490 | 0.0275 | 0.691 | 0.00999 | 1.90 |
|  | (0.617) | (0.439) | (0.830) | (0.0751) | (0.00776) | (0.000278) | (0.0341) | (0.00720) | (0.167) | (0.00270) | (0.234) |
| Other fruit | 8.42 | 3.17 |  | 0.465 | 1.54 | 0.571 | 1.64 | 2.10 | 2.70 | 0.861 | 0.171 |
|  | (0.329) | (0.180) |  | (0.0259) | (0.130) | (0.0390) | (0.0881) | (0.144) | (0.552) | (0.0430) | (0.0314) |
| Other vegetable | 1.90 | 2.18 | 0.0732 | 14.1 | 15.8 | 9.31 | 4.19 | 1.88 | 22.5 | 7.78 | 0.268 |
|  | (0.0529) | (0.0775) | (0.0211) | (0.409) | (0.341) | (0.369) | (0.200) | (0.0950) | (0.431) | (0.205) | (0.0227) |
| Roots tubers | 11.0 | 10.5 |  | 4.95 | 17.9 | 3.58 | 10.5 | 8.26 | 11.3 | 8.81 | 1.41 |
|  | (0.558) | (0.587) |  | (0.407) | (0.755) | (0.191) | (0.469) | (0.592) | (0.431) | (0.471) | (0.173) |
| Salty snacks & fried foods | 0.771 | 0.308 | 20.00 | 0.494 | 0.658 | 0.528 | 0.296 | 1.46 | 0.847 | 0.391 | 0.231 |
|  | (0.0505) | (0.0171) | (1.47) | (0.0258) | (0.0609) | (0.0264) | (0.0203) | (0.0875) | (0.153) | (0.0221) | (0.0223) |
| Sweets, confectionary | 1.57 | 1.80 | 11.6 | 2.01 | 5.37 | 1.94 | 1.28 | 4.58 | 1.40 | 1.97 | 3.87 |
|  | (0.190) | (0.195) | (0.74) | (0.123) | (0.750) | (0.103) | (0.157) | (0.267) | (0.612) | (0.229) | (0.428) |
| Vitamin A-rich fruits | 2.07 | 4.94 |  | 18.4 | 11.9 | 4.26 | 14.3 | 1.75 | 21.9 | 4.60 | 0.180 |
|  | (0.208) | (0.406) |  | (1.32) | (1.01) | (0.381) | (1.080) | (0.180) | (1.87) | (0.391) | (0.0280) |
| Vitamin A-rich vegetables tubers | 17.5 | 7.581 |  | 8.04 | 10.6 | 7.09 | 7.76 | 13.7 |  | 6.34 | 1.83 |
|  | (0.837) | (0.528) |  | (0.599) | (0.793) | (0.612) | (0.628) | (0.779) |  | (0.314) | (0.254) |
| **Observations** |  |  |  |  |  |  |  |  |  |  |  |
| Households | 6,102 |  |  |  |  |  |  |  |  |  |  |
| Food items | 129 |  |  |  |  |  |  |  |  |  |  |

^1^ Population statistics calculated using sampling weights. Standard errors in parentheses.
